# Understanding the theoretical underpinning of the exercise component in a fall prevention programme for older adults with mild dementia: a realist review protocol

**DOI:** 10.1186/s13643-016-0212-x

**Published:** 2016-07-19

**Authors:** Vicky Booth, Rowan Harwood, Victoria Hood, Tahir Masud, Philippa Logan

**Affiliations:** Division of Rehabilitation and Ageing, School of Medicine, University of Nottingham, Nottingham, NG7 2UH United Kingdom; Geriatric Medicine, Nottingham University Hospitals NHS Trust, Nottingham, NG7 2UH United Kingdom; School of Health Sciences, University of Nottingham, Nottingham, NG5 1PB United Kingdom

**Keywords:** Realist review, Realist synthesis, Accidental falls, Fall prevention, Exercise, Dementia, Cognitive impairment

## Abstract

**Background:**

Older adults with mild dementia are at an increased risk of falls. Preventing those at risk from falling requires complex interventions involving patient-tailored strength- and balance-challenging exercises, home hazard assessment, visual impairment correction, medical assessment and multifactorial combinations. Evidence for these interventions in older adults with mild cognitive problems is sparse and not as conclusive as the evidence for the general community-dwelling older population. The objectives of this realist review are (i) to identify the underlying programme theory of strength and balance exercise interventions targeted at those individuals that have been identified as falling and who have a mild dementia and (ii) to explore how and why that intervention reduces falls in that population, particularly in the context of a community setting. This protocol will explain the rationale for using a realist review approach and outline the method.

**Methods:**

A realist review is a methodology that extends the scope of a traditional narrative or systematic evidence review. Increasingly used in the evaluation of complex interventions, a realist enquiry can look at the wider context of the intervention, seeking more to explain than judge if the intervention is effective by investigating why, what the underlying mechanism is and the necessary conditions for success. In this review, key rough programme theories were articulated and defined through discussion with a stakeholder group. The six rough programme theories outlined within this protocol will be tested against the literature found using the described comprehensive search strategy. The process of data extraction, appraisal and synthesis is outlined and will lead to the production of an explanatory programme theory.

**Discussion:**

As far as the authors are aware, this is the first realist literature review within fall prevention research and adds to the growing use of this methodology within healthcare. This synthesis of evidence will provide a valuable addition to the evidence base surrounding the exercise component of a fall intervention programme for older adults with mild dementia and will ultimately provide clinically relevant recommendations for improving the care of people with dementia.

**Systematic review registration:**

PROSPERO CRD42015030169

**Electronic supplementary material:**

The online version of this article (doi:10.1186/s13643-016-0212-x) contains supplementary material, which is available to authorized users.

## Background

### Falls in older adults with MCI

Cognitive impairment is a risk factor for falls [1]. When an adult is cognitively impaired, regardless of the diagnosis, they are at increased risk of falls compared with age-matched cognitively intact individuals [2]. Mild cognitive impairment (MCI) is defined by measureable memory loss or other cognitive decline in the absence of interference with daily function [3]. Mild cognitive impairment is often a precursor to further cognitive deterioration and affects between 3 and 19 % of the population within the UK [4]. Every year, an estimated 15 % will go on to develop dementia [4]. Dementia is a syndrome of progressive and usually irreversible loss of memory and other cognitive functions including agnosia, apraxia, language and executive function, caused by a variety of brain diseases, and severe enough to interfere with daily function [5]. Mild dementia is an earlier stage within the clinical descriptors of dementia progression [6]. With the numbers of older adults diagnosed with dementia predicted to increase [7], falls in this population are likely to increase and fall prevention interventions will become more necessary. The intent of this is review is to focus on mild dementia populations and those with mild cognitive problems, such as MCI, that are theoretically on the cusp of rapid decline but whom are still cognitively able enough to engage and learn.

### Fall interventions

Fall prevention is a complex intervention that is well evidenced in a healthy older adult population. Through meta-analysis, exercise has shown to have consistent effects at reducing falls when prescribed and completed at the correct progression and intensity [8]. Many interventions have been trialled within populations who fall. Interventions in community-dwelling populations found to be effective include balance-challenging exercise, home hazard assessment and correcting visual impairment caused by cataract, as well as interventions directed at specific medical problems and multifactorial combinations [9]. In hospital and care home settings, the evidence is not as conclusive, with multifactorial fall prevention demonstrating positive but not statistically significant reductions in falls [10]. These meta-analyses identified numerous studies (156 and 60, respectively). National Institute of Clinical Excellence (NICE) guidelines recommend a multidisciplinary approach to fall prevention, with individualised assessment and programme prescription including strength and balance training [11]. Cognitive assessment is advised, but there is no guidance on how to respond to individuals with cognitive impairment. Recommendations and evidence for effective fall prevention interventions for older adults with cognitive impairment are not as well documented.

A recent systematic review by Guo et al. [12] identified 111 fall prevention studies of which only 12 had a homogenous sample of individuals with cognitive impairment. Their conclusion that exercise could be beneficial for those with cognitive impairment was based on the results from only one trial. A systematic review from 2013 [13] also identified small numbers of trials with only cognitively impaired adults (7 out of 11 included trials); they concluded that in this population there was as much evidence against exercise as there was for it. Both reviews highlighted heterogeneity amongst the samples due to differing cognition levels.

There is a strong rationale for early fall prevention in adults with mild cognitive problems as this population is at a higher risk of falling than people without impairment [14]. This population is at high risk of functional decline. Typical age of diagnosis of dementia is about 80 [15], and co-morbidity is common. Falls can contribute to this decline through injury, hospital admission, loss of confidence and deconditioning through reduced activity. If an intervention can reduce the risk of future falls at an early stage, then there is potential to maintain function and activity and reduce the progression into disability and dependency common in the fall population. In addition, there is evidence that neuroplastic adaptation in the brain can occur within a mild cognitively impaired population [16]. By helping people to adopt techniques to stay healthy (i.e. strength and balance exercises) and adaptations which reduce risk (i.e. mobility aids, home hazard reduction) at an early stage of cognitive impairment, these practices could theoretically then continue if further cognitive decline is experienced. Intervention is unlikely to reverse overall physical or cognitive functional problems. However, stabilising or slowing decline may help “compress morbidity” [17], by deferring deterioration by a year of two.

Before developing a suitable intervention, a review of the published literature was needed and a realist review methodology was chosen. The first stage of the review articulates the key theories and states how the review was conducted.

### Justification for this review

Historically, older adults with cognitive impairment were excluded from fall prevention trials to preserve a homogenous population sample and for practical and pragmatic trial management reasons. Anecdotally, this population has been associated with increased attrition and poor adherence due to memory and executive problems and co-morbidity. In the attempt to produce valid results, older adults with cognitive impairment have been excluded, leading to a lack of quantity and quality of evidence on fall interventions for this population. Whilst it is reasonable to assume that some findings from research conducted in healthy older populations can be extrapolated to older adults with dementia, differences are likely and not all findings will be relevant. Older adults with mild dementia have specific impairments (such as altered attentional capacity) that may influence the effectiveness of an intervention developed for a different patient population.

A realist review is a methodology that extends the scope of a traditional narrative or systematic evidence review. There are several reasons for choosing this method for investigations in this field. Firstly, a traditional systematic review focuses on the effectiveness of a particular intervention, and whilst this is useful, there are currently not enough studies in this patient population to determine the effectiveness of strength and balance exercise programmes with any certainty. Secondly, at this early stage of intervention development and available evidence, the theoretical underpinning of an intervention should be considered [18]. It is likely that adherence, context, barriers and facilitators, and the social and physical environment are important mediators of effectiveness, and a complete explanation must take them into account. Knowing what works for whom, how and why will direct the evolution of effective interventions. These are questions that a traditional systematic review does not ask. In his 2006 description of realist perspectives, Pawson outlined a critique of meta-analytical methods of the traditional systematic review, particularly in that by attempting to reduce bias from included studies “the very features that explain how interventions work are eliminated from the reckoning” (see p. 43 in [19]). Thirdly, there is substantial variability within dementia populations, such as the level of impairment (mild to severe) or dementia sub-type (Alzheimer’s disease, vascular, mixed, Lewy body, fronto-temporal dementia, etc.). Considering the limited literature available within this field, standard meta-analysis stratification is difficult and yields inconclusive results [9, 10, 12, 13]. A realist review allows consideration of different contextual variances. Fourthly, some important studies used methods that would not be included in a standard systematic review [20]. A realist review [21] allows the incorporation of detail from a range of literature with respect to the underlying processes and mechanisms of how an intervention reduces falls in a particular population. Realist enquiry has been recommended by the Medical Research Council within a process evaluation [22] to allow consideration of context and theory generation within intervention development, specifically when studying complex interventions and patient populations [23].

A realist review explores how underlying mechanisms (M) might be “triggered” in the context (C) of different intervention strategies to produce a reduction in falls or other outcomes (O). Within a realist enquiry, CMO configurations are interlinked and dependent upon each other, creating chains of conceptual possibilities or realities. Mechanisms are further subdivided between resources and responses. For example, a fall (O) could be prevented if an individual uses a stick (M^resource^) to feel safer (M^response^) when walking outside (C). This configuration is only relevant in the context of the individual walking outside and having the response of feeling safer by holding a stick.

The aim of the realist review is “explanation building” [14], providing a “contextualised understanding of how and why complex interventions achieve particular effects” ([19, p. 2]). Increasingly used in the evaluation of complex interventions, the realist enquiry can look at the wider context of the intervention, seeking more to explain than judge if the intervention is effective but investigating why, what the underlying mechanism is and the necessary conditions for success. It does this through definition of the “programme theories” or explicit theories and models of how an intervention achieves the desired or observed outcomes. A full definition of all terms is provided within the glossary.

Rough programme theories will be outlined, tested and refined against the literature through the review phases in a documented process.

### Objectives and focus of the review

The objective of the review is (i) to identify the underlying programme theory of strength and balance exercise interventions targeted at those individuals that have been identified as falling and who have a mild dementia and (ii) to explore how and why that intervention reduces falls in that population, particularly in the context of a community setting.

### Research questions

The aim of the current research is to understand how a strength and balance exercise programme might work with older adults with a cognitive impairment. In particular, what sorts of exercises reduce falls in older adults with cognitive impairment, under what circumstances, to what extent, and why? The research questions for the review are:

- What strength and balance exercises have been used in mild cognitive impairment populations to reduce falls?
- Why do those exercises reduce falls in that population?
- In what context do those exercises reduce falls in that population and to what extent?

## Methods

### Study design

The design utilises five practical stages of the review process identified by Pawson et al. [24]. This is not linear, and the reviewer moves between stages to achieve “theoretical saturation” (when no further information or findings have emerged against which to judge the programme theory) [21]. These stages include (i) articulating key rough programme theories to be explored, (ii) searching for relevant evidence, (iii) appraising quality of evidence, (iv) extracting the data and (v) synthesising evidence [24].

#### Articulation of key theories

Fall prevention is a complex intervention with many components that are introduced or employed according to individual patient characteristics. To clarify the scope of the review, the “patient journey” was identified, from case identification to discharge [8]. The underlying assumption is that through correct case identification and multidisciplinary assessment, multifactorial interventions identify and address risk factors causing falls. Initially, each stage of the “patient journey” was considered as sub-theories within an overall programme theory, containing possible CMO configurations relevant to that stage. The CMO configurations (CMOcs) were progressively refined to determine multiple rough programme theories within only one stage of the patient journey, the strength and balance exercises. This was an iterative process based on prior knowledge of the literature in fall prevention [25] and previous clinical experience. Mental models [26] were mapped according to potential CMOs and collated together into CMOcs. These initial rough theories were discussed with key stakeholders in fall prevention, including a patient and public representative, geriatricians, researchers and clinicians within the field. A stakeholder group involving these individuals was established. Consultation and discussion with this group was completed throughout this initial stage via a series of facilitated meetings and email discussion chains. Notes from the meetings were taken, and the refinement process for the rough programme theories was documented in paper format to ensure transparency. The researcher (VB) was also guided by the literature from relatively unstructured, exploratory internet-based searches. These were performed using broad search terms including the following: falls, cognitive impairment, intervention and prevention. A dialogue was maintained with the stakeholder group throughout this initial stage to ensure the researcher was maintaining relevance and accuracy to clinical practice.

The above process identified six rough programme theories to test against the literature. These were:

(A) Physiological changes

An older adult with dementia (C) completing a strength and balance exercise programme (M^resource^) will experience a physiological response (M^response^) which improves their physical ability (O^1^) and reduces their risk of falls (O^2^).

The hypothesised physiological responses are:

- Motor system: stronger muscles, quicker motor response, longer endurance, less fatigue, better control and coordination of muscle synergy
- Sensory system: improved proprioception
- Postural control: improved postural muscle activation and maintenance, quicker and more appropriate balance strategies (ankle, hip or stepping strategy) and response to perturbation
- Cognition: increased capacity to divide and maintain attention, improved visuospatial awareness, neuroplastic adaptations and changes

(B) Enjoyment

An older adult with dementia who has had a previously positive experience of falls or community services or exercise, who has a positive belief in exercise and who has the physical capability to do the exercises either independently or with support (C) will feel enjoyment (M^response^) from doing (O^1^) a strength and balance exercise programme (M^resource^), and this will reduce their risk of falls (O^2^).

(C) Encouraged (positive reinforcement)

An older adult with dementia who may not identify themselves as at risk of falling or remember any previous falls has poor or limited physical functioning, is not used to doing exercise but who is being appropriately supported (physically or emotionally) by a therapist (M^resource2^) or network (M^resource3^) and is well briefed or educated by that therapist or network (C) will feel encouraged (M^response1^) to do (O^1^) the strength and balance exercise programme (M^resource1^) and will recognise the positive benefits from participating in the programme (O^2^).

Once an older adult with dementia recognises the positive benefits from participating in a strength and balance exercise programme (C^2^), they will feel motivated (M^response2^) to continue with the programme (O^3^) and will reduce their risk of falls (O^4^).

(D) Fearful of negative consequences

An older adult with dementia who identifies themselves as at risk of falls remembers that they have previously fallen or had a “near miss”, has limited physical activity or function or ability and believes that they may deteriorate either physically or cognitively (C) or who listens to the education or warnings of the therapist (M^resource2^) will feel fearful (M^response1^) or concerned (M^response2^) and will do (O) the strength and balance exercise programme (M^resource1^).

(E) Empowered to achieve goal

An older adult who has something they want to achieve, whose goals align with that of the therapist (M^resource2^) and who believes that their goals can be achieved with the strength and balance exercise (M^resource1^) programme (C) will feel empowered (M^response^) to do (O^1^) the strength and balance exercise and achieve their goal (O^2^).

(F) Influenced by social and cultural expectations or beliefs

An older adult with dementia who believes that exercise is good, associates exercise with youth or vigour or health and well-being, who believes that the therapists or doctors “know what is best for them” (M^resource2^) and has a network that reinforces or imposes these beliefs (C) will feel influenced (M^response^) to do (O) the strength and balance exercise programme (M^resource1^).

**Fig. 1:**
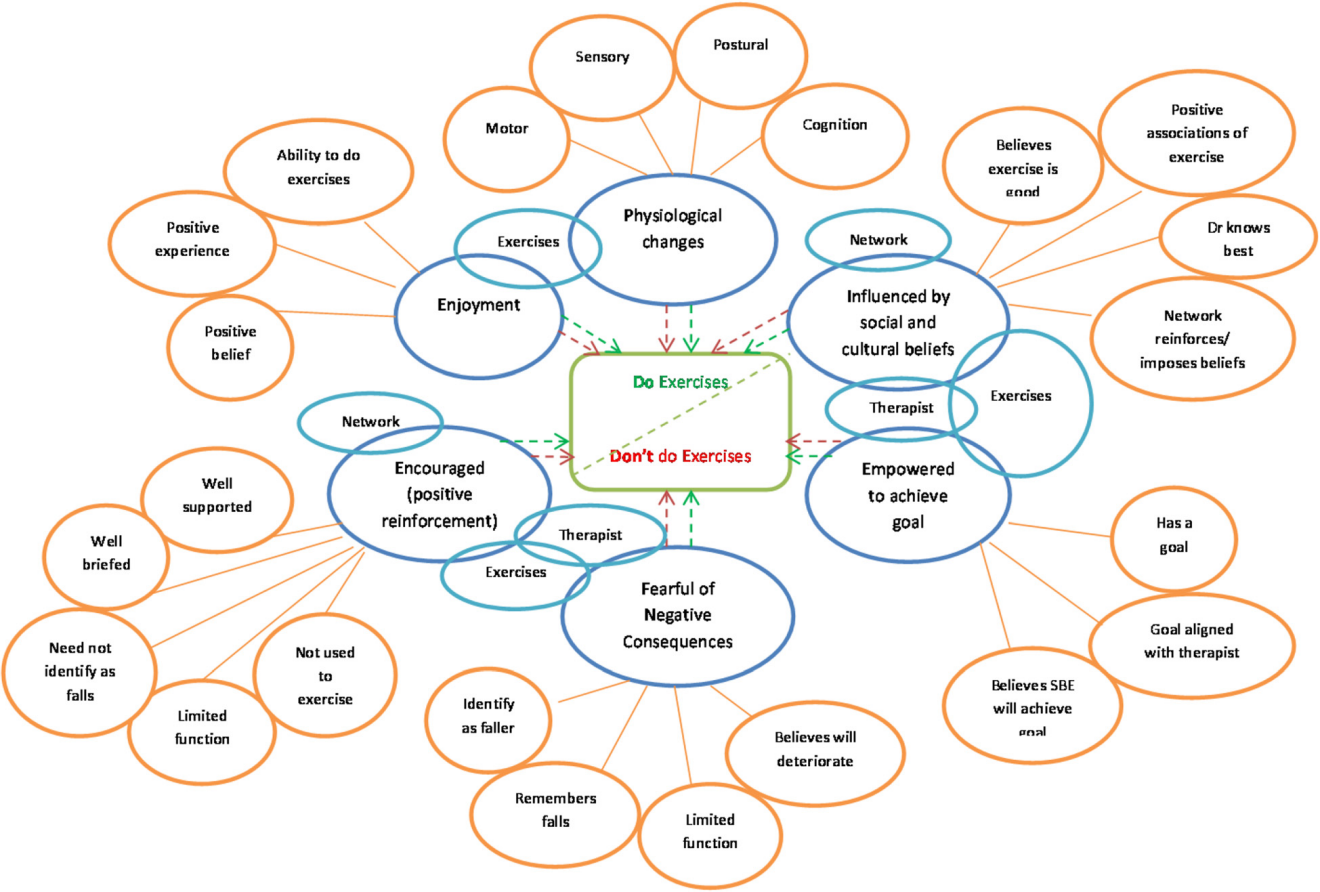
Diagram of rough programme theories. Legend: *Orange* = contexts, *Blue* = mechanisms (*light blue* = resources, *dark blue* = responses), *Green* = outcome

These rough programme theories are displayed in Fig. 1 in diagrammatical format.

#### Searching for relevant evidence

To capture all relevant material, a two-phased literature search will be conducted. The search will initially focus on exercise-based fall interventions in adults with mild cognitive problems to capture specific primary studies involving the intended population by using key phrases and words specific to the rough programme theories to test and refine them.

The search strategy will be as follows:

1. Electronic search of databases: EMBASE, MEDLINE, CINAHL, the Cochrane Library, PsycINFO, and PEDro. Keywords will be refined during the initial stage of the review process, but its thought will involve the MeSH terms *accidental falls*, *falls rehabilitation*, *exercise*, *dementia* and *cognitive impairment*. The search terms will be adapted according to the database used.
2. Electronic “cited by” search using Google Scholar.
3. Hand search of the reference lists of included papers.
4. Electronic search of the grey literature: EThoS and Google Scholar.

The second search phase will seek additional primary evidence that is specifically relevant to the testing and refinement of the rough programme theory. Material may be used that does not directly refer to older adults with cognitive impairment or fall prevention to assist consolidation of the programme theories (for example, material relating to people with Parkinson’s disease, traumatic brain injury or learning disability, or to activity or behaviour change in people with dementia). Additional searching may be completed if more data are required to refine a particular aspect of the programme theory that could not be evidenced or tested from the literature found within the initial and second search phase.

The searching will be both “iterative and interactive” [21], and it is therefore expected that the search terms will evolve as the searches are undertaken. Traditional keyword searching will be undertaken. The search results will be screened by the researcher (VB) who will document the number of articles retrieved during each search stage. EndNote reference management software will be used to track electronic documents and references.

#### Source selection

Selection of material for inclusion in the review will be based on relevance to the research aims and will provide information to test or assist in programme theory development. The articles will be considered for relevance based on an assessment of the “fit” of the article to the research question. The titles and abstracts of retrieved studies will be screened against pre-determined questions to ascertain the relevance of the material to the synthesis aims [27]. These will be:

1. For screening article titles: Could this be about the strength and balance exercise component of fall rehabilitation in older adults with mild cognitive problems in the community?
2. For screening article abstracts: Could this material provide useful information about completing the strength and balance exercise component of fall rehabilitation in older adults with mild cognitive problems in the community?

The inclusion of material will not be limited by document, article or study type and could include the following: trials, editorials, experimental studies, qualitative research, treatment manuals or evaluations. Material will be excluded if it is not published in English, does not involve older adults and does not involve community-based participants or interventions (such as those based within a hospital setting). Reasons for exclusion will be documented. If the reviewer is unsure about a document’s inclusion, then the second reviewer (PL or RH) will be used to aid decision-making. Disagreements between inclusion and relevance of material will be discussed with the stakeholder group. Material that is considered irrelevant for full-text retrieval will be kept until the end of the review process as once the initial stage of the search has been completed there may be relevant material to inform programme theory development and testing from studies initially dismissed due to topic specificity. For example, material that does not involve older adults but those with traumatic brain injury or learning disabilities that relates to a specific area of programme theory development might be considered for inclusion during the second search phase if required.

The full-text for eligible studies will be retrieved and assessed for quality and extraction of data by the main researcher (VB). Within realist synthesis and the quality standards developed for its reliability of reporting, there is no requirement for the screening, quality appraisal or data extraction to be completed independently by two researchers. Unlike a traditional systematic review, the realist process allows for theory development to be influenced from the material identified [21]. However, to ensure that the researcher is maintaining focus and consistency of judgement, a random sample of 10 % of the materials screened for inclusion will be selected and assessed by the second review author with the remaining 90 % completed by one reviewer (VB).

#### Appraising the quality of evidence

Quality appraisal and data extraction will be conducted simultaneously within the review process but are distinct and separate processes. Relevance and rigour are the two key processes within a realist synthesis when considering the quality appraisal and extraction of data [28] and will be used to ensure all selected materials should be included within the synthesis.

“A series of judgements” (see p. 35 in [28]) will be made concerning the rigour of the material found and the relevance of that material to answering the aims of the synthesis. As such, a combined data extraction sheet has been developed (Additional file 1). The rigour of the material must be sufficient to be included within the review itself. This will be judged by asking if “the methods used to generate the relevant data are credible and trustworthy” (see p. 35 in [28]). Rather than a technical checklist to appraise rigour [29], the reviewer will consider the credibility and trustworthiness using questions involving:

1. Is the material cohesive? Does it tell a comprehensive story or is there a juxtaposition of ideas or isolated statements?
2. What is the value of the evidence?
3. What is the material’s position in relation to the programme theory and general topic area?

As within a traditional systematic review, the judgements about the rigour of methods used to generate data that is relevant to the review are assessed prior to inclusion.

#### Extracting the data

Data will be extracted from the included material based on its relevance to the aims of the synthesis and the rough programme theories. Data is sought that either refines, substantiates or refutes the theories. Relevant material within the documents will be highlighted, labelled and documented on the data extraction sheet (Additional file 1). NVivo software will be used to record and code the extracted data.

During this stage of the review process, it may be necessary to conduct another search for materials if those found initially cannot build or test the rough programme theories. The researcher (VB) in collaboration with the stakeholder group will revisit and refine the search strategy, before conducting a purposive search with the revised focus.

#### Synthesising evidence

A series of questions will be asked of the extracted material to aid evidence synthesis. The data extraction sheet, adapted from previous realist reviews [30], outlines these questions (see Additional file 1), and include sections on:

1. Relevance
2. Interpretation of meaning
3. Judgements about CMO configurations (CMOcs)
4. Judgements about programme theory
5. Rigour
6. Population contextual information

The relevance of the text is questioned in relation to the rough programme theory. The content is then interpreted into context, mechanism or outcome relevancy. The CMOcs from the extracted material are written out, and judgements regarding their configurations are detailed. This will allow new CMOcs to be identified as well as compare the extracted material to the existing rough programme theories. Whether the material is trustworthy and rigorous enough to make changes to the rough programme theory or its CMOcs is then considered. Synthesis of the materials will occur in an iterative, complimentary process; as the researcher (VB) is engaging with and extracting data, there will be a simultaneous development of opinions and conclusions. A process of reasoning will occur whilst the questions from the data extraction sheet are being asked [19]. This reasoning process has been used in other realist syntheses [31] and includes:

- Juxtaposition of sources of evidence (for example, when evidence about implementation in one source enables insights into evidence about outcomes in another source)
- Reconciling of sources of evidence (when results differ in apparently similar circumstances, further investigation is appropriate in order to find explanations for why these different results occurred)
- Adjudication of sources of evidence (on the basis of methodological strengths or weaknesses)
- Consolidation of sources of evidence (when evidence about mechanisms and outcomes is complementary and enables a multifaceted explanation to be built)
- Situating sources of evidence (when outcomes differ in particular contexts, an explanation can be constructed of how and why these outcomes occur differently)

Extracted, primary data will be compared to the relevant rough programme theory (Fig. 1) to test and refine each pre-identified CMOc. Using the aforementioned reasoning categories, the final programme theory will be compiled concerning the context, mechanisms and corresponding outcomes for completing a strength and balance exercise programme as part of a fall intervention in older adults with mild cognitive problems. Each included study will be compared with the rough programme theories, with the relevant sections of text copied onto the data extraction sheet (Additional file 1).

The final programme theory will be narratively described, using text, tables and graphics as required. The review will be completed in accordance with guidance from the quality standards for a realist synthesis [29]. The results of the review will be reported as a series of clinical recommendations, for example, under these contexts (C), “x” is likely to happen (O) because of “y” (M). The recommendations will be context sensitive. The number and detail of recommendations will be based on the data extracted and the final programme theory. The final programme theory and CMOc recommendations will be narratively reported.

### Reporting and dissemination of findings

On completion, the review will be published in a leading journal within the field and the results presented at conference. The review will be reported according to the Realist and Meta-narrative Evidence Syntheses: Evolving Standards (RAMESES) publication standards [29] and the PRISMA-P statement (included as Additional file 2) [32]. The review will form a chapter of the PhD thesis of the researcher (VB) and therefore be available via eThesis from the host institution. A summary of the findings will be included in a report to the funding organisations (Alzheimer’s society on behalf of the Healthcare Management Trust). It is important that the findings of the review, if indicating valuable information for clinical practice within the area, are disseminated widely and with a clinical focus. This review will also be the first use of realist review methods in fall prevention in older adults with dementia; therefore, the process as well as the review findings will be included in any presentation or dissemination process.

### Ethical issues

Ethical approval will not be required to conduct this review. Consent and agreement of discussion documentation was sought from the stakeholder group regarding the development of the rough programme theories. Prior to funding being issued, the project was reviewed by peer and lay members of the Alzheimer Society and is continued to be monitored by an expert lay member panel. The review has been registered with PROSPERO (CRD42015030169).

## Discussion

### Limitations

This is the first realist literature review within fall prevention research, so it is important to highlight the limitations. Firstly, the methodology and theoretical assumptions behind realist enquiry encourages transparency about the influence of the researcher on certain aspects of the review, such as the initial rough programme theory development, and interpretation of material. Within this review, the researcher is clearly identified and the influences, whilst considered from a traditional systematic review method as producing potential for bias, assist the theory development in the review. As a clinician that has worked with this intervention in this patient population, there is potential for detailed recognition of the “hidden” mechanisms and understanding of the CMOcs. The stakeholder group and second reviewer are placed to ensure consistency and transparency of decision-making and to maintain the clinical and academic relevance of the review.

Secondly, the focus and scope of the review is limited by time and resources. The review is one chapter of a PhD thesis and is therefore constrained to be focused and relevant to the direction and aims of the whole PhD research project. It could be construed that this may hamper the detail and depth that the review can achieve.

Lastly, the published materials within this field may not include the detail of theoretical reasoning required to adequately test and define the programme theories. Research into falls is heavily weighted towards quantitative methods with publications following a rigid reporting structure. Calls for greater detail in publications regarding intervention reporting (such as the TiDieR guidelines [33]) may produce more context or resource information. However, it is expected that both the search strategy and subject field will need to be adapted as the review progresses (see search strategy).

### Summary

Falls within older adults with mild cognitive problems are important to both the health and wealth of the nation. Traditional systematic reviews report a dearth of evidence for fall interventions for this population. However, there is potential to consider the evidence from a different methodological perspective, to gain a better contextual and detailed picture of how these interventions are implemented, who they are being effective with and why. This synthesis of evidence will provide a valuable addition to the evidence base surrounding the exercise component of a fall intervention programme for older adults with mild dementia and will ultimately provide clinically relevant recommendations for improving the care of people with dementia.

## Consent for publication

Not applicable.

## Additional files

Additional file 1: Data analysis and synthesis process form. This combined data extraction sheet was developed specifically for this realist review. Additional file 2: PRISMA-P (Preferred Reporting Items for Systematic review and Meta-Analysis Protocols) 2015 checklist. Recommended items to address in a systematic review protocol.

## Abbreviations

CMO: context-mechanism-outcome
CMOc: context-mechanism-outcome configuration
MCI: mild cognitive impairment
NICE: National Institute of Clinical Excellence
RAMESES: Realist and Meta-narrative Evidence Syntheses: Evolving Standards
UK: United Kingdom

## Glossary

Context: The context describes anything in the social or physical world, such as the environment, the history of the programme or individual, cultural norms and values, beliefs or attitudes. The context is related to the mechanism, in that only those components which influence, trigger or modify the behaviour of a mechanism are relevant.
Context-mechanism-outcome configuration (CMOc): A CMO configuration is the relationship between particular contexts, mechanisms and outcomes. It can be reported as a statement, diagram or drawing. A sentence would be structured as so: “In ‘X’ context, ‘Y’ mechanism generates ‘Z’ outcome.”
Mechanism: It is widely acknowledged that mechanisms are difficult to define, have many definitions and are the most debated aspect within the field of realist enquiry. In this review, mechanisms generate outcomes, they are context-specific and sensitive and are often hidden or not observable [34]. Mechanisms can be divided into resources and responses.
Outcome: The outcome is the effect from the mechanism, in the particular contextual situation. It can be intended or unintended, positive or negative.
Programme theory: “A programme theory is an explicit theory or model of how an intervention, such as a project, a program, a strategy, an initiative, or a policy, contributes to a chain of intermediate results and finally to the intended or observed outcomes.” (see p. xix in [26]).
Rough programme theory: The rough programme theory is the starting theory to be tested against the literature during the review process. It is generated from the CMOcs and is therefore related to specific context characteristics but can also be generalisable across subject fields or domains. It is expected that the rough programme theory will develop and change as the review progresses. Rough programme theories are similar to candidate programme theories, a term used in other realist enquiry methods.

## References

[1] Lord SR, Sherrington C, Menz HB, Close JC (2007). Falls in older people: risk factors and strategies for prevention.

[2] Shaw FE, Bond J, Richardson DA, Dawson P, Steen IN, McKeith IG (2003). Multifactorial intervention after a fall in older people with cognitive impairment and dementia presenting to the accident and emergency department: randomised controlled trial. BMJ.

[3] Petersen RC (2003). Mild cognitive impairment: aging to Alzheimer’s disease.

[4] Ritchie K (2004). Mild cognitive impairment: an epidemiological perspective. Dialogues Clin Neurosci.

[5] Dening T, Thomas A (2013). Oxford textbook of old age psychiatry.

[6] Reisberg B (1987). Functional assessment staging (FAST). Psychopharmacol Bull.

[7] Alzheimer’s Society. Dementia 2014: opportunity for change. https://www.alzheimers.org.uk/infographic.

[8] Sherrington C, Tiedemann A, Fairhall N, Close JC, Lord SR (2011). Exercise to prevent falls in older adults: an updated meta-analysis and best practice recommendations. N S W Public Health Bull.

[9] Gillespie LD, Robertson MC, Gillespie WJ, Sherrington C, Gates S, Clemson LM (2012). Interventions for preventing falls in older people living in the community. Cochrane Database Syst Rev.

[10] Cameron ID, Gillespie LD, Robertson MC, Murray GR, Hill KD, Cumming RG (2012). Interventions for preventing falls in older people in care facilities and hospitals. Cochrane Database Syst Rev.

[11] NICE. Clinical Guideline 161. Falls: assessment and prevention of falls in older people https://www.nice.org.uk/guidance/cg161 [19/12/2014].

[12] Guo JL, Tsai YY, Liao JY, Tu HM, Huang CM (2014). Interventions to reduce the number of falls among older adults with/without cognitive impairment: an exploratory meta-analysis. Int J Geriatr Psychiatry.

[13] Winter H, Watt K, Peel NM (2013). Falls prevention interventions for community-dwelling older persons with cognitive impairment: a systematic review. Int Psychogeriatr.

[14] Taylor ME, Ketels MM, Delbaere K, Lord SR, Mikolaizak AS, Close JC (2012). Gait impairment and falls in cognitively impaired older adults: an explanatory model of sensorimotor and neuropsychological mediators. Age Ageing.

[15] Brayne C, Matthews FE, McGee MA, Jagger C (2001). Health and ill-health in the older population in England and Wales The Medical Research Council Cognitive Function and Ageing Study (MRC CFAS). Age Ageing.

[16] Belleville S, Clement F, Mellah S, Gilbert B, Fontaine F, Gauthier S (2011). Training-related brain plasticity in subjects at risk of developing Alzheimer’s disease. Brain.

[17] Fries JF (1980). Aging, natural death, and the compression of morbidity. N Engl J Med.

[18] Craig P, Dieppe P, Macintyre S, Michie S, Nazareth I, Petticrew M (2008). Developing and evaluating complex interventions: the new Medical Research Council guidance. BMJ.

[19] Pawson R (2006). Evidence-based policy: a realist perspective.

[20] Halvarsson A, Dohrn M, Ståhle A (2014). Taking balance training for older adults one step further: the rationale for and a description of a proven balance training programme. Clin Rehabil.

[21] Pawson R, Greenhalgh T, Harvey G, Walshe K (2004). Realist synthesis: an introduction.

[22] Moore GF, Audrey S, Barker M, Bond L, Bonell C, Hardeman W (2015). Process evaluation of complex interventions: Medical Research Council guidance. BMJ.

[23] Greenhalgh T, Wong G, Westhorp G, Pawson R (2011). Protocol--realist and meta-narrative evidence synthesis: evolving standards (RAMESES). BMC Med Res Methodol.

[24] Pawson R, Greenhalgh T, Harvey G, Walshe K (2005). Realist review–a new method of systematic review designed for complex policy interventions. J Health Serv Res Policy.

[25] Booth V, Logan P, Harwood R, Hood V (2015). Falls prevention interventions in older adults with cognitive impairment: a systematic review of reviews. International Journal of Therapy & Rehabilitation.

[26] Funnell SC, Rogers PJ (2011). Purposeful program theory: effective use of theories of change and logic models.

[27] Blane DN, Macdonald S, Morrison D, O’Donnell CA (2015). Interventions targeted at primary care practitioners to improve the identification and referral of patients with co-morbid obesity: a realist review protocol. Systematic reviews.

[28] Wong G, Westhorp G, Pawson R, Greenhalgh T (2013). Realist synthesis. RAMESES training materials.

[29] Wong G, Greenhalgh T, Westhorp G, Buckingham J, Pawson R (2013). RAMESES publication standards: realist syntheses. BMC Med.

[30] Wong G, Brennan N, Mattick K, Pearson M, Briscoe S, Papoutsi C (2015). Interventions to improve antimicrobial prescribing of doctors in training: the IMPACT (IMProving Antimicrobial presCribing of doctors in Training) realist review. BMJ open.

[31] Brennan N, Bryce M, Pearson M, Wong G, Cooper C, Archer J (2014). Understanding how appraisal of doctors produces its effects: a realist review protocol. BMJ open.

[32] Shamseer L, Moher D, Clarke M, Ghersi D, Liberati A, Petticrew M (2015). Preferred reporting items for systematic review and meta-analysis protocols (PRISMA-P) 2015: elaboration and explanation. BMJ.

[33] Hoffmann TC, Glasziou PP, Boutron I, Milne R, Perera R, Moher D (2014). Better reporting of interventions: template for intervention description and replication (TIDieR) checklist and guide. BMJ.

[34] Astbury B, Leeuw FL (2010). Unpacking black boxes: mechanisms and theory building in evaluation. Am J Eval.

