# Supplementary material for: Understanding the theoretical underpinning of the exercise component in a fall prevention programme for older adults with mild dementia: a realist review protocol
Source: Syst Rev. 2016 Jul 19;5:119. doi: 10.1186/s13643-016-0212-x (PMC4952275; doi:10.1186/s13643-016-0212-x)
Supplement: Additional file 1: — Data analysis and synthesis process form. This combined data extraction sheet was developed specifically for this realist review. [file 13643_2016_212_MOESM1_ESM.docx]

**Supplementary Material**

**Data Analysis and Synthesis Process form**

| **Reference:** | | | |
| --- | --- | --- | --- |
|  | | | |
| **1. Relevance:**  *Are the contents of a section of text within an included document referring to data that might be relevant to our programme theories? Which ones?* | | | |
| A. Physiological changes:  B. Enjoyment:  C. Encouraged (positive reinforcement):  D. Fearful of negative consequences:  E. Empowered to achieve goal:  F. Influenced by social and cultural beliefs: | | | |
|  | | | |
| **2. Interpretation of meaning:**  *If it is relevant, do the contents of a section of text provide data that may be interpreted as being context, mechanism (resource/response) or outcome?* | | | |
|  | | | |
|  | | | |
| **3. Judgements about Context-Mechanism-Outcome-Configurations:**  *What is the Context-Mechanism (resource)-Mechanism (response)-Outcome Configuration (CMOC) (partial or complete) for the* ***data****?* | | | |
| **Context** | **Resource/Intervention** | **Response/Mechanism** | **Outcome** |
|  |  |  |  |
|  |  |  |  |
|  |  |  |  |
|  | | | |
| **4. Judgements about programme theory:**   - *How does this (full or partial) CMOC relate to the programme theory?* - *Within this same document are there data which informs how the CMOC relates to the programme theory?* - *If not, are these data in other documents? Which ones?* - *In light of this CMOC and any supporting data, does the programme theory need to be changed? How?* | | | |
|  | | | |
|  | | | |
| **5. Rigour:**   - *Are the data sufficiently trustworthy and rigorous to warrant making changes to the CMOC?* - *Are the data sufficiently trustworthy and rigorous to warrant making changes to the programme theory?* | | | |
|  | | | |
|  | | | |
| **6. Contextual Information:** | | | |
| - Age: - Cognitive status/level: - Intervention setting: - Intervention details: | | | |
|  | | | |
| **Notes:** | | | |
|  | | | |
