# Supplementary material for: Understanding the theoretical underpinning of the exercise component in a fall prevention programme for older adults with mild dementia: a realist review protocol
Source: Syst Rev. 2016 Jul 19;5:119. doi: 10.1186/s13643-016-0212-x (PMC4952275; doi:10.1186/s13643-016-0212-x)
Supplement: Additional file 2: — PRISMA-P (Preferred Reporting Items for Systematic review and Meta-Analysis Protocols) 2015 checklist. Recommended items to address in a systematic review protocol. [file 13643_2016_212_MOESM2_ESM.doc]

**PRISMA-P (Preferred Reporting Items for Systematic review and Meta-Analysis Protocols) 2015 checklist: recommended items to address in a systematic review protocol***

| Section and topic | Item No | Checklist item | Page |
| --- | --- | --- | --- |
| ADMINISTRATIVE INFORMATION | | | |
| Title: |  |  |  |
| Identification | 1a | The report is identified as a protocol of a systematic review | 1 |
| Update | 1b | n/a the protocol is not an update of a previous systematic review | - |
| Registration | 2 | The review is registered with PROSPERO (CRD42015030169) | 3 |
| Authors: |  |  |  |
| Contact | 3a | The name, institutional affiliation, and e-mail address of all protocol authors is provided with the physical mailing address of corresponding author | 1-2 |
| Contributions | 3b | The contributions of protocol authors is described | 20 |
| Amendments | 4 | n/a the protocol is not an amendment of a previously completed or published protocol | - |
| Support: |  |  |  |
| Sources | 5a | Sources of financial support for the review have been identified | 20 |
| Sponsor | 5b | The review funder and sponsor have been provided | 20 |
| Role of sponsor or funder | 5c | The role of external support in developing the protocol has been provided | 20 |
| INTRODUCTION | | | |
| Rationale | 6 | The rationale for the review in the context of what is already known is described | 3-7 |
| Objectives | 7 | An explicit statement of the question(s) the review will address with reference to participants, interventions, comparators, and outcomes (PICO) is provided | 8 |
| METHODS | | | |
| Eligibility criteria | 8 | The study characteristics to be used as criteria for eligibility for the review are described in accordance with realist review publication standards | 13-15 |
| Information sources | 9 | All intended information sources are described | 12 |
| Search strategy | 10 | The draft of search terms are provided but in keeping with realist review methodology the development of the search strategy will occur following the initial search | 12 |
| Study records: |  |  |  |
| Data management | 11a | The mechanism that will be used to manage records and data throughout the review is described | 15 |
| Selection process | 11b | The study selection process is described including the involvement of a second reviewer to corroborate data extracted and theory generation | 13-15 |
| Data collection process | 11c | The planned method of extracting data from reports (including data extraction and synthesis form) is provided | 15-17 |
| Data items | 12 | The data items relate to the rough programme theory which has been described in detail | 9-11 |
| Outcomes and prioritization | 13 | The outcomes for which data will be sought relate to the rough programme theories described within the protocol, there is no prioritization and this is not explained in alignment with previous realist reviews | 9-11 |
| Risk of bias in individual studies | 14 | n/a as realist reviews consider the relevance and rigour of included studies, this process is described | - |
| Data synthesis | 15a | The criteria under which study data will be synthesised is described | 15-17 |
|  | 15b | n/a data will not be extracted for quantitative synthesis | - |
|  | 15c | n/a data will not be extracted for additional analyses (such as sensitivity or subgroup analyses, meta-regression) | - |
|  | 15d | n/a data will not be extracted for quantitative synthesis therefore alternative synthesis methods are not appropriate | - |
| Meta-bias(es) | 16 | n/a the realist review is not considering any planned assessment of meta-bias(es) (such as publication bias across studies, selective reporting within studies) | - |
| Confidence in cumulative evidence | 17 | The strength of the body of evidence will be determined using a series of judgements from the review authors in alignment with previous realist reviews and realist review publication standards | 15-17 |

*** It is strongly recommended that this checklist be read in conjunction with the PRISMA-P Explanation and Elaboration (cite when available) for important clarification on the items. Amendments to a review protocol should be tracked and dated. The copyright for PRISMA-P (including checklist) is held by the PRISMA-P Group and is distributed under a Creative Commons Attribution Licence 4.0.**

*From: Shamseer L, Moher D, Clarke M, Ghersi D, Liberati A, Petticrew M, Shekelle P, Stewart L, PRISMA-P Group. Preferred reporting items for systematic review and meta-analysis protocols (PRISMA-P) 2015: elaboration and explanation. BMJ. 2015 Jan 2;349(jan02 1):g7647.*
